# The Question of Rest for Women during Menstruation

**Published:** 1877-08

**Authors:** 


### Books Reviewed.

---

*The Question of Rest for Women During Menstruation.* By MARY PUTNAM JACOBI, M. D., Professor of Materia Medica in the Woman's Medical College, New York.
*The Boylston Prize Essay of Harvard Universitiy, for* 1876. Illustrated. New York: G. P. Putnam's Sons, 182 Fifth Avenue, 1877. Buffalo: Peter Paul & Bro. pp. 232.

The writer of this essay interrogates the processes of nature in a candid and philosophical manner for the answer to the question at issue. To this interrogation she comes with the strength of profound erudition, abundant resources with which to prosecute her inquiry, a practice of many years, access without limit to large hospitals and learned societies, the wealth of immense libraries, a disposition for research and unquestioned ability. These should concur to the formation of opinions worthy of respect. The conclusion is given on page 227 in the following words: "There is nothing in the nature of menstruation to imply the necessity or even the desirability of rest for women *whose nutrition* is *really normal.* This is not however a mere opinion, but the summing up of a demonstration, of which the reader will get some idea from the quotations that follow. A table of statistics is prepared from the written statements of two hundred and sixty-eight women of different social class and rank, with varying occupations and ladies of leisure. This statement covers the whole period of conscious life up to the time it is made, also the condition of family health, &c., very much after the fashion of life insurance, but is on the whole more minute in detail, and covers a wider range of possibilities. The age at which school life commenced, number of hours spent each day in school, kind of education pursued, whether common, ornamental or higher, number of hours spent in study out of school and number of hours habitually passed in recreation, are noted also the general condition of health and presence or absence and character of pain during before and after menstruation. Strength is measured in this estimate by capacity for exercise in walking. Condition with regard to size, color, weight &c., is also taken into account. Ninety-four persons record themselves as never having suffered either pain, discomfort or weakness during the menstrual flow. This is 35 per cent of the whole. The total number of cases is then divided into two classes. Those who suffer and those who do not are separately analyzed. The results are tabulated in nearly every conceivable manner for convenience of comparison. On page 62 the final summing up of this investigation is given in the following words, "Judging by these statistics alone, therefore, we should say that immunity from menstrual suffering was to be expected in proportion to:

1. The vigor of health during childhood and the soundness of family history, especially in regard to freedom from constitutional taint of scrofula, consumption, or rheumatism, or family tendency to uterine disease.

2. To the degree of exercise taken during school life.

3. To the thoroughness and extension of the mental education.

4. To the degree to which general health and capacity for exercise were maintained after cessation of study.

5. To steadiness of occupation.

6. Finally to marriage at a suitable time."

On page 97 "The sum of notions in regard to the processes involved in menstruation that have been acquired in the last few years by Waldeyer" and others, are contrasted in columns with those formulated by Pouchet and those adopting the ovulation theory proper. The writer of this essay announces her belief in the Waldeyer school as opposed to the ovulation theory. On page 115, the author says "On the hypothesis that the menstrual period represents the climax in the development of a surplus of nutritive force and material we should expect to find a rhythmic wave of nutrition gradually rising from a minimum point just after menstruation to a maximum just before the next flow. The traces of this rhythmic wave should be measured by the consumption of oxygen and the excretion of $CO_2$, and of urea; by the tension of the arterial system, the vital capacity of the lungs, possibly also by the dynamic force of the muscles." Then follow tables showing the results of daily measurements of urea, average of pulse and temperature, and variations of muscular force as measured by a hand dynamometer. Where the test was made by lifting weights the diminution was most uniform. Traces by Mahomed's sphygmograph obtained from eight individuals, and taken inter, pre and post menstrually are presented in a series of plates. The author says on page 164, "In all the details examined therefore we find evidences of such a gradual but steady preparation for the menstrual hemorrhage as should exclude the idea that this *when normal* has any tendency to deplete the nutrition or lower the strength." On page 225 "When persons in good health suffer severe menstrual pain, this is nearly always dependent upon some anatomical imperfection of the uterus. When persons in delicate health are free from menstrual distress it is because the supplemental wave of nutrition does not rise sufficiently to greatly increase general vascular tension, or because the uterus is too slightly hyperæmiated to excite reaction." Page 230 "Whenever women exhibit mental irritability and consequent weakness at, or before, mensturation, it is a proof that the resistance of their nerve centres is weakened, below the normal standard, sometimes congenitally and by inheritance. In these cases rest is desirable during whatever period of the month the nervous excitement may be experienced, but this will be more frequently through the two or three days preceding the hemorrhage, than at the time of the flow. In slighter cases rest is unnecessary ; in those more severe it is in itself useless, i. e. other measures must be taken in order to cure the disease. In those cases of congenital hysteria where a cure can not be effected, it is evident that the women *are* permanently unfit to bear severe responsibility or to undergo mental strain."

American women are individually and collectively placed under a debt of gratitude to Dr. Jacobi for the able and thorough investigation she has made of this subject and the fearless manner in which she has written. The Dr. has done more than to answer the question. If women whose nutrition is not normal require labor and rest alternated at shorter intervals than do those in a normal condition, this is also true of all conditions of innutrition without regard to age or sex. We have extended this notice beyond the limits allotted to us on account of the immense interests capable of being affected by false or true opinions with regard to this subject. Books more or less recently written for popular reading have taken false positions and disseminated false opinions, thereby creating the greater need for the genuinely able and scientific investigation and elucidation which we now have in this volume. M. B. M.

